# Curcumin Prevents Aflatoxin B_1_ Hepatoxicity by Inhibition of Cytochrome P450 Isozymes in Chick Liver

**DOI:** 10.3390/toxins8110327

**Published:** 2016-11-10

**Authors:** Ni-Ya Zhang, Ming Qi, Ling Zhao, Ming-Kun Zhu, Jiao Guo, Jie Liu, Chang-Qin Gu, Shahid Ali Rajput, Christopher Steven Krumm, De-Sheng Qi, Lv-Hui Sun

**Affiliations:** 1 Department of Animal Nutrition and Feed Science, College of Animal Science and Technology, Huazhong Agricultural University, Wuhan 430070, China; zhangniya@mail.hzau.edu.cn (N.-Y.Z.); qi_ming1993@126.com (M.Q.); ling930910@126.com (L.Z.); zhumingkun1989@126.com (M.-K.Z.); guojiao1991@sina.com (J.G.); cherishlj@webmail.hzau.edu.cn (J.L.); dr.shahidali@hotmail.com (S.A.R.); qds@mail.hzau.edu.cn (D.-S.Q.); 2 College of Veterinary Medicine, Huazhong Agricultural University, Wuhan 430070, China; guchangqin@mail.hzau.edu.cn; 3 Department of Animal Science, Cornell University, Ithaca, NY 14853, USA; csk97@cornell.edu

**Keywords:** curcumin, aflatoxin B_1_, CYP450, AFBO–DNA, chicks

## Abstract

This study was designed to establish if Curcumin (CM) alleviates Aflatoxin B_1_ (AFB_1_)-induced hepatotoxic effects and to determine whether alteration of the expression of cytochrome P450 (CYP450) isozymes is involved in the regulation of these effects in chick liver. One-day-old male broilers (*n* = 120) were divided into four groups and used in a two by two factorial trial in which the main factors included supplementing AFB_1_ (< 5 vs. 100 μg/kg) and CM (0 vs. 150 mg/kg) in a corn/soybean-based diet. Administration of AFB_1_ induced liver injury, significantly decreasing albumin and total protein concentrations and increasing alanine aminotransferase and aspartate aminotransferase activities in serum, and induced hepatic histological lesions at week 2. AFB_1_ also significantly decreased hepatic glutathione peroxidase, catalase, and glutathione levels, while increasing malondialdehyde, 8-hydroxydeoxyguanosine, and exo-AFB_1_-8,9-epoxide (AFBO)-DNA concentrations. In addition, the mRNA and/or activity of enzymes responsible for the bioactivation of AFB_1_ into AFBO—including CYP1A1, CYP1A2, CYP2A6, and CYP3A4—were significantly induced in liver microsomes after 2-week exposure to AFB_1_. These alterations induced by AFB_1_ were prevented by CM supplementation. Conclusively, dietary CM protected chicks from AFB_1_-induced liver injury, potentially through the synergistic actions of increased antioxidant capacities and inhibition of the pivotal CYP450 isozyme-mediated activation of AFB_1_ to toxic AFBO.

## 1. Introduction

Aflatoxins (AF) are secondary fungal metabolites that are largely produced by the fungi *Aspergillus flavus* and *Aspergillus parasiticus* [1,2]. Among the various dangerous AF and their metabolites, aflatoxin B_1_ (AFB_1_) is the most toxic, exhibiting harmful hepatotoxic, teratogenic, mutagenic, and carcinogenic effects on humans and many species of livestock [3,4,5,6]. It is also classified as a Group I carcinogen [7]. Human or animal consumption of the food or feed contaminated by AFB_1_ can pose serious problems to their health and productivity, and thus result in significant economic losses [8,9]. The toxic effects of AFB_1_ are associated with its toxification and detoxification biotransformation pathways. Upon being delivered to the liver, AFB_1_ is bioactivated by cytochrome P450 (CYP450)—a member of the phase I metabolizing enzymes—into the highly reactive exo-AFB1-8,9-epoxide (AFBO) [3,10]. AFBO can form adducts with DNA and other critical macromolecules, causing toxicity, mutations, and cancer [10]. Meanwhile, AFB_1_ can induce the generation of reactive oxygen species (ROS), which can lead to oxidative stress, potentially mediated via CYP450 activity [11,12]. On the other hand, AFBO can be detoxified via conjugation with glutathione (GSH) to form a non-toxic adduct, which can be catalyzed by glutathione-S transferases (GSTs), the phase II detoxification enzymes [10].

Curcumin (CM) is a natural polyphenolic compound extracted from rhizomes of *Curcuma longa* Linn (turmeric), widely used as household spice, natural food colorant, and herbal medicine in many Asian countries for thousands of years [13]. It possesses antioxidant, anti-inflammatory, radio-protective, chemotherapeutic, anti-cancer, and detoxification abilities in laboratory animals and humans [14,15,16,17]. Previous publications have described that CM can effectively mitigate AFB_1_-induced adverse effects in several animal species [6,15,18,19,20]. Moreover, the protective action of CM against AFB_1_-induecd adverse effects was further demonstrated by improving the antioxidant capacity [18,19,21,22]. Notably, as different CYP450 isozymes catalyze AFB_1_ to various metabolites, including the highly toxic AFBO and the less- or non-toxic aflatoxicol, AFM_1_, AFP_1_, and AFQ_1_, examination of the regulation of the proportions of CYP450 isozymes by CM would be valuable [10,23]. Previous studies have described that CM can alter various CYP450 isozymes in vivo and in vitro [24,25,26,27]. Moreover, CM inhibition of AFB_1_ toxicity has been reported by modulating CYP450 function [14], while the knowledge of which crucial CYP450 isozymes are involved in this process remains unknown. Chicken orthologs of human CYP1A, 2A, and 3A families are the main CYP450 enzymes responsible for the bioactivation of AFB_1_ into the highly toxic AFBO in chicken [23]. However, there is limited information on the effect of CM on these pivotal CYP450 isozymes that are involved in AFB_1_ metabolism. Therefore, we selected chickens to investigate whether dietary supplementation of CM mitigated AFB_1_-induced hepatotoxic effects though the regulation of these key CYP450 isozymes.

## 2. Results

### 2.1. Growth Performance, Serum Biochemistry, and Liver Histology

**Figure 1:**
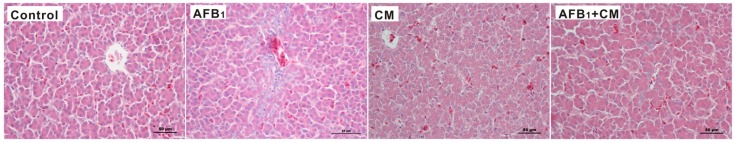
Photomicrographs of hepatic sections stained with hematoxylin and eosin (400× magnification) of chicks from different treatment groups at week 2. AFB_1_, aflatoxin B_1_; CM, curcumin. Experimental details of Control and AFB_1_ groups are given in Sun et al. (2016) [12].

Non-significant differences in average daily feed intake, average daily gain, and feed/gain ratio were observed among the four groups throughout the experiment (Table S1). Serum biochemical parameters were significantly affected by either supplementation of AFB_1_ or CM at week 2 (Table 1). Compared to the control, the activities of alanine aminotransferase (ALT) and aspartate aminotransferase (AST) increased (*p* < 0.05) by 33.3% and 43.8% respectively, while the concentrations of albumin (ALB) and total protein (TP) decreased (*p* < 0.05) by 33.8% and 26.0%, respectively, in the serum of chicks by AFB_1_ supplementation. Notably, the serum biochemical parameter changes observed in the AFB_1_ group were prevented in the AFB_1_ + CM group. However, no significant differences in these serum biochemical parameters were observed among the four groups at week 4 (Table 1). Since the serological results indicated AFB_1_ only induced liver injury at week 2, we selected samples from week 2 to explore the mechanism. Furthermore, dietary AFB_1_ exposure induced liver injury as shown through bile duct hyperplasia and necrosis at week 2. Strikingly, the AFB_1_ + CM group prevented the hepatic injury observed in the AFB_1_ group (Figure 1).

### 2.2. Hepatic Antioxidant Parameters and CYP450 Isozyme Activities

After 2 weeks of experimental treatments, the antioxidant parameters and CYP450 isozyme activities were significantly altered by either supplementation of AFB_1_ or CM (Table 2 and Table 3). Compared to the control, supplementation of dietary AFB_1_ led to a decrease (0.05) in the activities of glutathione peroxidase (GPX, 13.1%), catalase (CAT, 16.2%), and GSH concentration (30.9%), along with increase (*p <* 0.05) in concentrations of malondialdehyde (MDA, 100.0%) and 8-hydroxydeoxyguanosine (8-OHdG, 17.9%) in the liver of chicks at week 2, respectively. Interestingly, the antioxidant parameter changes observed in the AFB_1_ group were prevented in the AFB_1_ + CM group (Table 2). In addition, supplementation of CM alone increased (*p <* 0.05) activity of GPX (25%), while it did not affect the other antioxidant parameters, when compared with the control. Meanwhile, the dietary AFB_1_ supplementation led to an increase (*p <* 0.05) in the activity of CYP1A1 (270.6%), CYP1A2 (99.4%), CYP2A6 (184.5%), and CYP3A4 (29.2%) in the liver microsomes of chickens, respectively (Table 3). Strikingly, the increased CYP450 isozyme activities observed in the AFB_1_ group were reduced in the AFB_1_ + CM group.

### 2.3. Hepatic AFBO–DNA Adduct Contents

**Figure 2:**
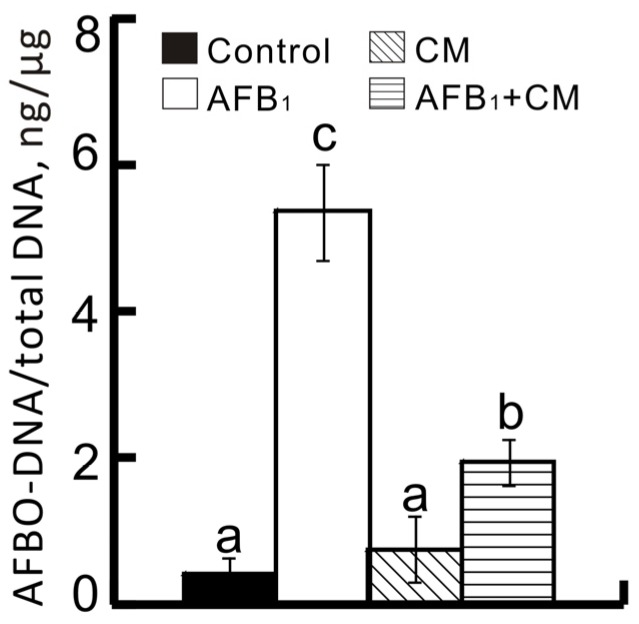
Effects of dietary AFB_1_ and CM concentrations on the contents of AFBO–DNA adducts in the liver of chicks at week 2. Values are expressed as means ± SD (*n* = 5), and means with different superscript letters differ (*p* < 0.05). AFB_1_, aflatoxin B_1_; AFBO, exo-AFB1-8,9-epoxide; CM, curcumin. Experimental details of Control and AFB1 groups are given in Sun et al. (2016) [12].

The concentrations of AFBO–DNA adducts in the liver were significantly affected by either supplementation of AFB_1_ or CM at week 2 (Figure 2). Compared to the control, the hepatic AFBO–DNA adduct content was increased (*p <* 0.05) 12 times by AFB_1_ supplementation. Interestingly, the AFB_1_ + CM group decreased (*p <* 0.05) the concentration of AFBO–DNA adduct (63.7%) in the liver when compared to the AFB_1_ group.

### 2.4. Hepatic CYP450 Isozyme Activities and Gene Expression

**Figure 3:**
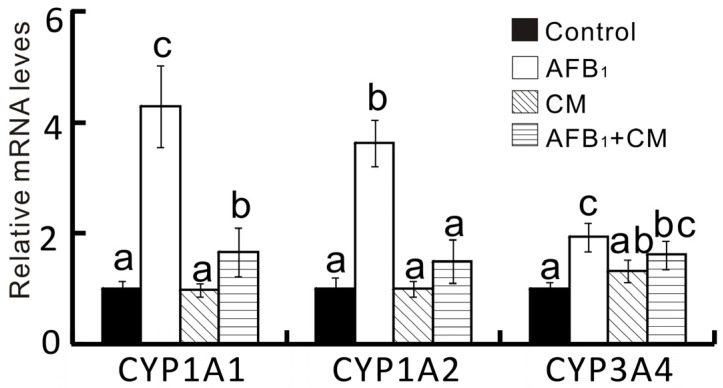
Effects of dietary AFB_1_ and CM concentrations on relative mRNA abundance of CYP450 isozyme genes in liver of chicks at week 2. Values are expressed as means ± SD (*n* = 5), and means with different superscript letters differ (*p* < 0.05). AFB_1_, aflatoxin B_1_; CM, curcumin; *CYP1A1*, Cytochrome P450 1A1; *CYP1A2*, Cytochrome P450 1A2; *CYP3A4*, Cytochrome P450 3A4. Experimental details of Control and AFB1 groups are given in Sun et al. (2016) [12].

The mRNA levels of *CYP1A1*, *CYP1A2*, and *CYP3A4* in the liver were significantly altered by either supplementation of AFB_1_ or CM (Figure 3). Specifically, dietary AFB_1_ supplementation led to upregulated (*p <* 0.05) mRNA levels of *CYP1A1*, *CYP1A2*, and *CYP3A4* in liver microsomes. Strikingly, the increased hepatic CYP450 isozyme mRNA levels observed in the AFB_1_ group were suppressed in the AFB_1_ + CM group. It is fascinating to find that the effects of AFB_1_ and CM on changes in hepatic CYP450 isozyme mRNA levels were in parallel with their activities.

## 3. Discussion

Protection against AFB_1_-induced hepatotoxic effects was successfully replicated in broiler chicks fed an AFB_1_-contaminated corn–soybean diet with CM supplementation. Although the dietary AFB_1_ had no significant effect on growth performance in chicks, it induced the typical clinical signs of hepatic injury, including increased activities of AST and ALT, decreased concentrations of ALB and TP in serum, as well as bile duct hyperplasia and necrosis in the liver of chicks at week 2 [28,29]. However, the serological results indicated that AFB_1_-induced liver injury disappeared at week 4. The reasons for this might be that older poultry was more resistant to aflatoxicosis than young poultry [30], and the AFBO–DNA adducts could be repaired by the nucleotide excision repair system in liver [31]. Intriguingly, dietary supplementation of CM mitigated serum and histopathological parameter alterations that were induced by dietary supplementation of AFB_1_ at week 2. These outcomes were consistent with previous studies, which provided evidence that hepatic injury was induced by dietary AFB_1_ as well as that dietary CM supplementation displayed protective effects against its negative effects [15,19,20,21]. Moreover, the present study displayed AFB_1_-induced oxidative stress in chickens as evidenced by decreased antioxidant ability (GPX, CAT, and GSH), increased lipid peroxidation (MDA), and DNA damage (8-OHdG). On the other hand, dietary CM supplementation inhibited these changes. Meanwhile, dietary supplementation of CM alone improved the hepatic GPX activity, which was consistent with previous studies in which CM increased GPX activity, probably by activating the Nrf2–keap1 pathway [32,33]. A previous study showed that dietary supplementation of 5000 mg/kg CM increased hepatic phase II detoxification enzyme (GST) activity in rats that were exposed to AFB_1_ [34], while GST activity was not affected by CM in this study. The divergence between these reports could be attributed to the different animal species and ingestion dose. Taken together, these outcomes are similar to former studies, which reported that oxidative stress could be due to the direct effects of AFB_1_, its metabolites, and/or the generation of free radicals [11,35]. Dietary supplementation of CM, however, showed protective actions against AFB_1_-induced hepatic injury, which were associated with the enhancement of antioxidant capacities [18,19,21,22].

The most interesting finding from the present study was that the four major CYP450 isozymes were significantly inhibited to a large extent by dietary supplementation of CM upon exposure to dietary AFB_1_. The hepatic mRNA levels and/or enzyme activities of CYP1A1, CYP1A2, CYP2A6, and CYP3A4 were significantly increased when chicks were exposed to dietary AFB_1_, while dietary supplementation of CM inhibited these changes. Because a previous study reported that CYP2A6 and (to a lesser extent) CYP1A1 are responsible for the bioactivation of AFB_1_ into AFBO in chicken hepatic microsomes, and that CYP1A2 and CYP3A4 are the most important enzymes capable of bioactivating AFB_1_ into AFBO in mammals [23,36], inhibition of the activities of these enzymes could decrease the production of AFBO. Indeed, as a major toxic adduct of AFBO [10,36], the AFBO–DNA was sharply decreased by the dietary supplementation of CM when chicks were exposed to dietary AFB_1_. These findings suggest that the protective actions of CM may be mediated through inhibited activities of these crucial CYP450 isozymes, which could decrease the production of the highly toxic AFBO. These outcomes are in agreement with previous studies, which showed that CM-mediated inhibition of AFB_1_ toxicity was associated with a reduction of the formation of AFBO–DNA by modulating CYP450 function, including CYP1A1 activity [14,20], while the activities of CYP1A2, CYP2A6, and CYP3A4 inhibited by CM in this process are described, to our knowledge, for the first time in the current study. Similarly, CM has been shown to inhibit tumorigenesis induced by benzo(a)pyrene and 2,3,7,8-tetrachlorodibenzo-p-dioxin through the inhibition of CYP1A1, CYP1B1, and/or CYP1A2 enzyme on mRNA, protein, and/or activities levels [25,37].

The CYP450s are important metabolizing enzymes, most of which are mainly expressed in the liver. Clinically, these enzymes play vital roles in drug metabolism and are required for the efficient clearance of xenobiotics from the body [38,39]. On the other hand, these enzymes can also bioactivate biologically inert carcinogens and toxins, such as 4-(methylnitrosamino)-1-(3-pyridyl)-1-butanone [40], *N*-nitrosonornicotine [41], hexamethylphosphoramide [42], benzo(a)pyrene [25], AFB_1_ [10], and 2,6-dichlorobenzonitrile [43] to electrophilic metabolites that can cause toxicity, cell death, and sometimes cellular transformation that results in cancer. Given that CM is a strong inhibitor of CYP450 isozymes, it could be a potentially promising chemopreventive agent for many carcinogens and toxins, even though its mechanism of action remains to be clarified.

In summary, the present study successfully confirmed that dietary CM supplementation could alleviate AFB_1_-induced liver injury, with regard to the suppression of serum biochemistry changes and histopathological lesions in the liver of broilers. The protective mechanism of CM against AFB_1_-induced adverse effects may be associated with: (1) the common mechanism that CM could reduce AFB_1_-induced oxidative stress by increasing antioxidant capacities; and (2) the novel finding that CM could effectively inhibit the regulatory role of CYP450 isozymes that are crucial for the activation of AFB_1_ to highly toxic AFBO. Further validation of the potential mechanisms of the interactions between CM and the CYP450 isozymes would be beneficial to gain a better understanding of the detoxification mechanism of CM and applications of CM to control chemical carcinogenesis.

## 4. Materials and Methods

### 4.1. Chicks, Treatments, and Samples Collection

Our animal protocol was approved by the Scientific Ethic Committee of Huazhong Agricultural University on 8 August 2015. The project identification code is HZAUCH-2015-007. In total, 120 one-day-old male avian broilers were randomly divided into four treatment groups with five replicates of six chicks per pen. All chicks were allowed free access to a corn/soybean-based diet (BD) formulated to meet the nutritional requirements of broilers’ diets (Table S2) as reported previously [12], and distilled water. After three days of acclimation, the four experimental groups were arranged in a two by two factorial design trial that included BD diet supplemented with AFB_1_ (Sigma-Aldrich, St. Louis, MO, USA) and CM (China National Medicines Corp. Ltd., Beijing, China), as follows: Control; AFB_1_ (100 µg AFB_1_/kg); CM (150 mg CM/kg); and AFB_1_ + CM (100 µg AFB_1_/kg with 150 mg CM/kg). The experiment lasted for four weeks. The doses were chosen based on previous studies, which reported that dietary consumption of approximately 100 μg AFB_1_/kg induced adverse effects [44], while dietary supplementation of 74–222 mg CM/kg displayed a protective effect on AFB_1_ in broilers [19]. Individual body weights and feed intake of broilers were measured biweekly. Meanwhile, chicks (*n* = 5/group) were euthanized by decapitation to collect blood and livers for the preparation of serum and liver histological tissue samples as previously described [29].

### 4.2. Dietary AFB_1_ Analysis, and Feed Preparation

Twenty grams of moldy corn was extracted with 100 mL of methanol (Fisher, Pittsburgh, PA, USA):water (70:30, *v/v*) for AFB_1_ detection. After shaking for 3 min, the supernatant of the extract was filtered through a Whatman filter (Whatman, Clifton, NJ, USA), and the filtrate was collected. Then, the concentration of AFB_1_ in filtrate was measured followed the protocol of the ELISA kit (AgraQuant^®^ Aflatoxin B_1_ Assay, Romer, Singapore). The powdered feed was mixed with a vertical mixer.

### 4.3. Serum Biochemical and Histological Analysis

The serum activities of aspartate aminotransferase (AST) and alanine aminotransferase (ALT), along with concentrations of albumin (ALB) and total protein (TP) were determined in serum samples. Analysis of the serum samples was measured by an automatic biochemistry analyzer (Beckman Synchron CX4 PRO, Fullerton, CA, USA). The liver tissues were fixed in 10% neutral buffered formalin and processed for paraffin embedding, sectioned at 5 µm, and then stained with hematoxylin and eosin, by standard procedure [29]. Liver sections from all broilers were microscopically examined. 

### 4.4. Antioxidant Enzyme Activities Analysis

Liver samples (0.5 g) were thawed in 4.5 mL isotonic saline on ice and homogenized as previously described [33]. The supernatants were then prepared by centrifugation at 12,000 × *g* for 15 min at 4 °C. Activities of superoxide dismutase (SOD), glutathione peroxidase (GPX), catalase (CAT), and GST, as well as concentrations of GSH and malondialdehyde (MDA) were determined using the colorimetric method through the specific assay kits (A001, A005, A007-1, A004 A006-1 and A003), which were purchased from the Nanjing Jiancheng Bioengineering Institute of China. The concentration of 8-hydroxydeoxyguanosine (8-OHdG) in serum was measured using the ELISA kit (H165, Nanjing Jiancheng Bioengineering Institute, Nanjing, China). Concentrations of protein were determined using the bicinchoninic acid assay [45].

### 4.5. Hepatic AFBO–DNA Adduct Analysis

Liver genomic DNA was extracted using the DNA extraction kit following the manufacturer’s instructions (Qiagen, Shanghai, China). The DNA concentrations were quantified by the 260/280 nm absorbance ratio by an Agilent Bioanalyzer 2100 (Agilent Technologies, Amstelveen, The Netherlands). Genomic DNA (15 μg) was used to determine the AFBO–DNA adduct amount using a competitive ELISA method, according to the manufacturer’s instructions (Cell Biolabs, Inc., San Diego, CA, USA).

### 4.6. Hepatic Microsomal CYP450 Isozyme Activities Analysis

The liver microsomes and cytosolic fractions were prepared as described previously [46]. The microsomal activities of 7-ethoxyresorufin-*O*-deethylase, methoxyresorufin-*O*-demethylase, coumarin 7-hydroxylase, and nifedipine oxidation were determined to assess chicken orthologs of human CYP1A1, CYP1A2, CYP2A6, and CYP3A4 activities, respectively [23]. The concentrations of protein were measured as described above [45].

### 4.7. Real-Time Quantitative Polymerase Chain Reaction (qPCR)

Total RNA was extracted from liver samples by using Trizol (Invitrogen, Carlsbad, CA, USA) following the manufacturer’s instructions. The quality and quantity of the RNA samples were analyzed by an Agilent Bioanalyzer 2100 using an RNA 6000 Labchip kit (Agilent Technologies, Amstelveen, The Netherlands). The cDNA was synthesized from 1.0 μg total RNA by using Super Script III reverse transcriptase (Invitrogen). The mRNA levels of CYP450 isozyme genes were determined by qPCR (7900 HT; Applied Biosystems, Foster City, CA, USA). The primers of CYP450 isozymes and reference gene glyceraldehyde-3-phosphate dehydrogenase were reported previously [12]. The 2^−ddCt^ method was used for the quantification, with glyceraldehyde 3-phosphate dehydrogenase (*GAPDH*) as a housekeeping gene and the relative abundance normalized to the control (as 1) [47].

### 4.8. Statistical Analysis

One-way ANOVA followed by Fisher’s least significant difference (LSD) test was used to compare the effects between groups on each variable within the same tissue. Data are presented as means ± SD, and significance level was set at *p <* 0.05. All analyses were conducted using SAS 8.2 (SAS Institute, Cary, NC, USA).

## Figures and Tables

**Table 1:** Effects of dietary AFB_1_ and CM concentrations on serum biochemical parameters in chicks ^1^.

Item	Control	AFB1	CM	AFB_1_ + CM
Week 2				
ALT, U/L	1.2 ± 0.1 ^a^	1.6 ± 0.4 ^b^	1.2 ± 0.3 ^a,b^	1.3 ± 0.2 ^a,b^
AST, U/L	176.7 ± 27.0 ^a^	254.2 ± 53.9 ^b^	178.4 ± 38.1 ^a^	193.5 ± 39.4 ^a,b^
TP, g/L	17.7 ± 1.1 ^b^	13.1 ± 2.3 ^a^	17.3 ± 1.8 ^b^	19.1 ± 2.0 ^b^
ALB, g/L	7.4 ± 0.3 ^b^	4.9 ± 1.0 ^a^	7.1 ± 1.2 ^b^	8.1 ± 1.0 ^b^
Week 4				
ALT, U/L	1.0 ± 0.1	1.2 ± 0.3	1.4 ± 0.4	1.6 ± 0.5
AST, U/L	217.4 ± 26.8	211.2 ± 22.0	223.6 ± 37.5	245.9 ± 83.4
TP, g/L	22.8 ± 3.9	23.3 ± 2.7	21.8 ± 4.4	26.3 ± 7.8
ALB, g/L	9.9 ± 2.1	9.8 ± 1.9	9.3 ± 2.4	11.6 ± 3.7

^1^ Values are expressed as means ± SD (*n* = 5), and means with different superscript letters differ (*p* < 0.05). AFB_1_, aflatoxin B_1_; ALB, albumin; ALT, alanine aminotransferase; AST, aspartate aminotransferase; CM, curcumin; TP, total protein. Experimental details of Control and AFB_1_ groups are given in Sun et al. (2016) [12].

**Table 2:** Effects of dietary AFB_1_ and CM concentrations on hepatic antioxidant parameters in chicks at week 2 ^1^.

Item	Control	AFB_1_	CM	AFB_1_ + CM
GPX, U/mg	127.8 ± 5.1 ^b^	111.1 ± 10.3 ^a^	159.9 ± 15.1 ^c^	159.7 ± 8.9 ^c^
SOD, U/mg	156.8 ± 5.2 ^a,b^	149.9 ± 9.7 ^a^	170.2 ± 6.7 ^b^	162.5 ± 8.7 ^a,b^
CAT, U/mg	13.6 ± 0.9 ^b^	11.4 ± 1.6 ^a^	15.4 ± 1.2 ^b,c^	16.7 ± 1.0 ^c^
GST, U/mg	61.5 ± 1.1	60.6 ± 1.9	62.3 ± 5.1	62.0 ± 2.4
GSH, μmol/g	48.5 ± 10.1 ^b^	33.5 ± 3.9 ^a^	62.8 ± 17.6 ^b^	53.4 ± 15.6 ^b^
MDA, μmol/g	3.2 ± 0.4 ^a^	6.4 ± 1.3 ^b^	2.9 ± 0.5 ^a^	3.2 ± 0.6 ^a^
8-OHdG, nmol/mg	152.2 ± 8.1 ^a^	179.5 ± 5.4 ^b^	157.9 ± 2.9 ^a^	156.8 ± 4.7 ^a^

^1^ Values are expressed as means ± SD (*n* = 5), and means with different superscript letters differ (*p <* 0.05). AFB_1_, aflatoxin B_1_; CAT, catalase; CM, curcumin; GPX, glutathione peroxidase; GSH, glutathione; GST, glutathione-S transferases; MDA, malondialdehyde; SOD, superoxide dismutase; 8-OHdG, 8-hydroxydeoxyguanosine. Experimental details of Control and AFB_1_ groups are given in Sun et al. (2016) [12].

**Table 3:** Effects of dietary AFB_1_ and CM concentrations on the activities of chicken cytochrome P450 (CYP450) orthologs in the liver at week 2 ^1^.

Item	Control	AFB_1_	CM	AFB_1_ + CM
CYP1A1, nmol/(mgprotein·min)	0.34 ± 0.13 ^a^	1.26 ± 0.17 ^c^	0.83 ± 0.15 ^b^	0.79 ± 0.16 ^b^
CYP1A2, nmol/(mgprotein·min)	1.71 ± 0.45 ^a^	3.41 ± 0.49 ^b^	2.45 ± 0.63 ^a,b^	1.95 ± 0.55 ^a^
CYP2A6, nmol/(mgprotein·min)	2.07 ± 0.30 ^a^	5.89 ± 1.37 ^b^	3.00 ± 1.18 ^a^	3.08 ± 0.63 ^a^
CYP3A4, nmol/(mgprotein·min)	26.93 ± 2.22 ^a^	34.79 ± 3.06 ^b^	24.86 ± 1.51 ^a^	22.60 ± 2.26 ^a^

^1^ Values are expressed as means ± SD (*n* = 5), and means with different superscript letters differ (*p <* 0.05). AFB_1_, aflatoxin B_1_; CM, curcumin; CYP1A1, Cytochrome P450 1A1; CYP1A2, Cytochrome P450 1A2; CYP2A6, Cytochrome P450 2A6; CYP3A4, Cytochrome P450 3A4. Experimental details of Control and AFB_1_ groups are given in Sun et al. (2016) [12].

## Supplementary Materials

The following are available online at www.mdpi.com/2072-6651/8/11/327/s1, Table S1: Effects of dietary AFB_1_ and CM concentrations on growth performance in chicks, Table S2: Basal diet formulations and nutritional contents.
