# Supplementary material for: Curcumin Prevents Aflatoxin B1 Hepatoxicity by Inhibition of Cytochrome P450 Isozymes in Chick Liver
Source: Toxins (Basel). 2016 Nov 10;8(11):327. doi: 10.3390/toxins8110327 (PMC5127124; doi:10.3390/toxins8110327)
Supplement: Supplementary file 1 [file toxins-08-00327-s001.pdf]

# Supplementary Materials: Curcumin Prevents Aflatoxin $B_1$ Hepatotoxicity by Inhibition of Cytochrome P450 Isozymes in Chick Liver

**Ni-Ya Zhang, Ming Qi, Ling Zhao, Ming-Kun Zhu, Jiao Guo, Jie Liu, Chang-Qin Gu, Shahid Ali Rajput, Christopher Steven Krumm, De-Sheng Qi and Lv-Hui Sun**

**Table S1.** Effects of dietary $AFB_1$ and CM concentrations on growth performance in chicks [1].

| Item | Control | $AFB_1$ | CM | $AFB_1$ + CM |
|---|---|---|---|---|
| Week 1–2 | | | | |
| Body weight gain, g/day | 34.0 ± 1.4 | 34.9 ± 1.4 | 33.8 ± 2.0 | 33.4 ± 2.9 |
| Feed intake, g/day | 48.4 ± 8.3 | 46.8 ± 5.6 | 45.8 ± 5.5 | 46.1 ± 5.8 |
| Gain/feed, g/kg | 703 ± 132 | 767 ± 106 | 746 ± 83.2 | 734 ± 128 |
| Week 3–4 | | | | |
| Body weight gain, g/day | 68.3 ± 8.0 | 72.9 ± 8.7 | 68.2 ± 6.9 | 70.1 ± 6.3 |
| Feed intake, g/day | 116 ± 7.8 | 125 ± 22.9 | 111.9 ± 8.1 | 118 ± 11.6 |
| Gain/feed, g/kg | 607 ± 26.5 | 589 ± 27.4 | 609 ± 38.3 | 592 ± 41.3 |

[1] Values are expressed as means ± SD ($n$ = 5), and means with different superscript letters differ ($p$ < 0.05). AFB1, aflatoxin B1; CM, curcumin. Experimental details of Control and AFB1 groups are given in Sun et al. (2016) [1].

**Table S2.** Basal diet formulations and nutritional contents [1].

| Ingredients | Percentage (%) | Nutrition Component | Content |
|---|---|---|---|
| Corn | 58.2 | Crude protein (%) | 20.3 |
| Wheat bran | 2.0 | Metabolizable energy (MJ/kg) | 12.3 |
| Soybean meal | 30.0 | Calcium (%) | 1.0 |
| Soybean oil | 2.5 | Available phosphorus (%) | 0.43 |
| Cottonseed meal | 3.0 | Methione (%) | 0.60 |
| $CaCO_3$ | 1.0 | Methione+cystine (%) | 0.90 |
| CaHPO4 | 2.0 | Lysine (%) | 1.10 |
| Salt | 0.3 | Trytophan (%) | 0.22 |
| L-Lysine (%) | 0.15 | Threonine (%) | 0.69 |
| DL-Methione (%) | 0.3 | | |
| Choline chloride | 0.2 | | |
| Vitamin premix [2] | 0.05 | | |
| Mineral premix [3] | 0.3 | | |

[1] The 5% corn in basal diet was substituted by 5% moldy corn in $AFB_1$ and CM+$AFB_1$ group diets, respectively, which made the $AFB_1$ contents up to 100 ug/kg in these two diets. $AFB_1$, $AFB_1$, aflatoxin $B_1$; CM, curcumin. [2] Vitamin premix provided per kg of diet: retinyl acetate, 10,280 IU; cholecalciferol 2280 IU; dl-$\alpha$-tocopheryl acetate, 17.12 mg; menadione, 6.82 mg; thiamin, 2.28 mg; riboflavin, 5.68 mg; pantothenic acid, 12.25 mg; pyridoxine, 2.28 mg; niacin, 22.84 mg; biotin, 0.18 mg; folic acid, 1.12 mg. [3] Mineral premix provided per kg of diet: iron, 100 mg; copper, 8 mg; manganese, 20 mg; zinc, 100 mg; iodine, 0.7 mg.

## Reference

1. Sun, L.H.; Zhang, N.Y.; Zhu, M.K.; Zhao, L.; Zhou, J.C.; Qi, D.S. Prevention of Alfatoxin B1 hepatoxicity by dietary selenium is associated with inhibition of cytochrome P450 isozymes and up-regulation of six selenoprotein genes in chick liver. *J. Nutr.* **2016**, *143*, 1115–1122.
